# The Wnt/*β*-Catenin Signaling Pathway Controls the Inflammatory Response in Infections Caused by Pathogenic Bacteria

**DOI:** 10.1155/2014/310183

**Published:** 2014-07-17

**Authors:** Octavio Silva-García, Juan J. Valdez-Alarcón, Víctor M. Baizabal-Aguirre

**Affiliations:** Centro Multidisciplinario de Estudios en Biotecnología, Facultad de Medicina Veterinaria y Zootecnia, Universidad Michoacana de San Nicolás de Hidalgo, Km. 9.5 s/n Carretera Morelia-Zinapécuaro, La Palma, Tarímbaro, 58893 Morelia, MICH, Mexico

## Abstract

Innate immunity against pathogenic bacteria is critical to protect host cells from invasion and infection as well as to develop an appropriate adaptive immune response. During bacterial infection, different signaling transduction pathways control the expression of a wide range of genes that orchestrate a number of molecular and cellular events to eliminate the invading microorganisms and regulate inflammation. The inflammatory response must be tightly regulated because uncontrolled inflammation may lead to tissue injury. Among the many signaling pathways activated, the canonical Wnt/*β*-catenin has been recently shown to play an important role in the expression of several inflammatory molecules during bacterial infections. Our main goal in this review is to discuss the mechanism used by several pathogenic bacteria to modulate the inflammatory response through the Wnt/*β*-catenin signaling pathway. We think that a deep insight into the role of Wnt/*β*-catenin signaling in the inflammation may open new venues for biotechnological approaches designed to control bacterial infectious diseases.

## 1. The Wnt/*β*-Catenin Pathway

The protein *β*-catenin is a ubiquitously expressed main signal transducer of the canonical Wnt signaling pathway. It is also found in adherent junctions, forming a complex with E-cadherin, *α*-catenin, and actin filaments of the cytoskeleton [1]. The Wnt/*β*-catenin is an ancient and evolutionary conserved pathway present in lime molds, worms, and humans, with a prominent role in embryogenesis, cell differentiation, and stem cell maintenance self-renewal. Recently, mutations in genes encoding *β*-catenin or other Wnt pathway components have been identified in certain types of cancer, fibrosis, and inflammatory bowel disease [2].

**Figure 1:**
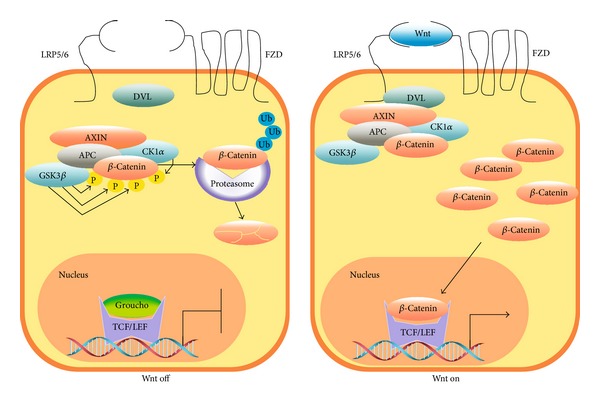
The Wnt/*β*-catenin pathway. In the absence of stimulus (Wnt off), *β*-catenin is constantly phosphorylated by CKI*α* and GSK3*β*. These phosphorylations constitute a signal for *β*-catenin polyubiquitination and hydrolysis by the proteasome 26S. In the presence of Wnt protein ligands (Wnt on), the* destruction complex *constituted by the proteins APC, Axin, CKI*α*, and GSK3 is inactivated and *β*-catenin, which is constantly synthetized, accumulates in the cytoplasm and nucleus where it interacts with TCF/LEF transcription factors to enhance expression of specific genes.

*β*-Catenin is not detectable in the cytoplasm or nucleus in unstimulated cells because free *β*-catenin levels (the form not bound to the E-cadherin complex) is controlled by the* destruction complex*. This complex is composed mainly by the tumor suppressor adenomatous polyposis coli (APC) and Axin, which function as scaffolding proteins on which the Ser/Thr kinases casein kinase 1*α* (CK1*α*) and glycogen synthase kinase 3*β* (GSK3*β*) phosphorylate *β*-catenin at the N-terminal residues Ser45, Ser33, Ser37, and Thr41 [3]. These phosphorylations label *β*-catenin to be polyubiquitinated by the Skp1-Cdc53-F-Box E3 ubiquitin ligase complex (SCF^*β*-TRCP^) and degraded by the proteasome 26S (Figure 1, Wnt Off). GSK3*α* is also able to regulate *β*-catenin phosphorylation* in vitro *and* in vivo*, which indicates that both isoforms of GSK3 contribute to maintain low levels of *β*-catenin in basal conditions [4].

The Wnt/*β*-catenin pathway is activated when a secreted Wnt glycoprotein binds to the N-terminal extracellular cysteine rich domain of a Frizzled (Fzd) receptor (Figure 1, Wnt On). There are 19 Wnt proteins and 10 Fzd receptors identified in humans, whose function, regulation, and interaction in different cellular processes are currently an active area of research. Upon binding of Wnt to Fzd, the coreceptor low density lipoprotein 5/6 (LRP5/6) interacts with Fzd and dishevelled (DVL), leading to the recruitment of APC and Axin to the plasma membrane along with CK1*α* and GSK3*β*. In turn, CK1*α* and GSK3*β* phosphorylate LRP5/6 in the PPPSP motif, decreasing the GSK3*β*-dependent phosphorylation of *β*-catenin through a substrate competitive mechanism. As *β*-catenin is continuously synthetized, Wnt-induced inactivation of the* destruction complex* causes a transient stabilization and accumulation of *β*-catenin in the cytoplasm. Then *β*-catenin translocates to the nucleus and, by displacing the corepressor Groucho, enhances the expression of specific genes through the interaction with the DNA-bound T cell factor/lymphoid enhancer factor (TCF/LEF) proteins. Gene expression ends when the transcription complex is disassembled and nuclear APC exports *β*-catenin to the cytoplasm where it is degraded [2].

Approximately 400 genes involved in cell growth, differentiation, apoptosis, survival, and immune functions are regulated by the Wnt/*β*-catenin signaling pathway. A proinflammatory role of Wnt/*β*-catenin has been recently documented in 3T3-L1 preadipocytes stimulated with Wnt1 [5]. Activation of Wnt/*β*-catenin pathway with Wnt3a in mouse microglial cells leads to the expression and release of the proinflammatory cytokines interleukins (IL)-6, IL-12, and IFN*γ* [6]. In contrast, an anti-inflammatory role of Wnt/*β*-catenin pathway has also been demonstrated in mouse colon epithelial stem cells and macrophages infected with* Salmonella* [7] or* Mycobacterium* [8], which indicates that activation of the Wnt/*β*-catenin pathway downregulates the proinflammatory responses to certain bacterial infections [9, 10]. These apparently contradictory results when cells are stimulated with either Wnt proteins or pathogenic bacteria suggest that the anti- or proinflammatory role of Wnt/*β*-catenin may depend on the stimulus, the cell type, the activation context, and its crosstalk with other signaling pathways. In the next sections, we discuss experimental data demonstrating that Wnt/*β*-catenin is an important signaling pathway in the regulation of bacterial-induced inflammatory response.

## 2. *Salmonella typhimurium*: Crosstalk with the NF-*κ*B Pathway

The role of Wnt/*β*-catenin in* Salmonella *infections has been investigated* in vitro *or in streptomycin pretreated mice models [7, 11–13]. From these studies it is known that infection of colon epithelial cells (CEC) with* Salmonella typhimurium* causes an increase in GSK3*β*-dependent *β*-catenin phosphorylation, leading to an upregulation of IL-6, IL-8, and Wnt2, via TLR5/NF-*κ*B activation [10, 14]. These results were confirmed in HCT116 and T84 cell lines by expressing constitutively active *β*-catenin mutants that, upon interaction with the NF-*κ*B p50 subunit, decreased the NF-*κ*B DNA binding and transcriptional activities [10]. Similar results were obtained in cells pretreated with LiCl, an inhibitor of GSK3 activity, which mimics activation of Wnt/*β*-catenin [14]. It is worth to mention that infection with* Salmonella *induced a decrease in Axin1, which is a scaffolding protein controlling the levels of *β*-catenin [15]. These data indicate that induction of the NF-*κ*B proinflammatory pathway by* Salmonella *is dependent upon *β*-catenin degradation, suggesting that *β*-catenin plays a negative regulatory role in the inflammatory response.

**Figure 2:**
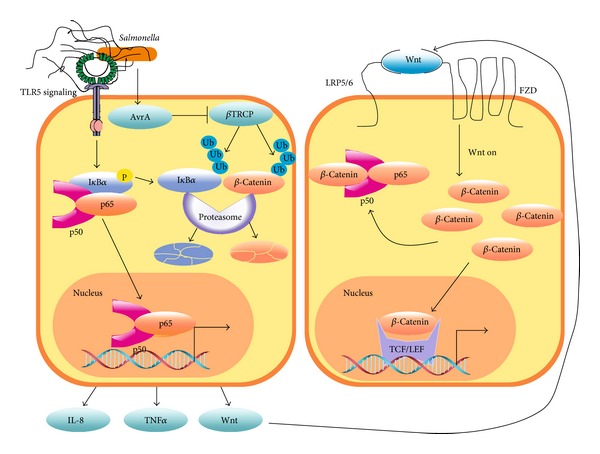
*β*-Catenin is an antagonist of the NF-*κ*B pathway. In colon epithelial cells,* Salmonella *induces IL-8, TNF*α*, and Wnt expression via NF-*κ*B activation. Moreover, the* Salmonella typhimurium *deubiquinating effector AvrA stabilizes I*κ*B*α* and *β*-catenin. This step enhances *β*-catenin activity and at the same time inhibits NF-*κ*B by stabilizing its inhibitor. The expression of Wnt in the first stage of infection stimulates Wnt/*β*-catenin pathway. The stabilized *β*-catenin then interacts with the NF-*κ*B p50 subunit, decreasing its transcriptional activity. While NF-*κ*B activity is inflammatory, its inhibition by *β*-catenin activation constitutes a mechanism to reduce inflammation.

Infection of CEC with the avirulent* Salmonella *Phop^c^ strain induced an increase of *β*-catenin phosphorylation at Ser552, a site associated with enhanced transcriptional activity, indicating that a protein effector is responsible for *β*-catenin stabilization [7]. In fact, the* Salmonella *effector protein AvrA has been shown to affect *β*-catenin stability because infection of CEC with the* Salmonella *strain SL1344 Phop^c^ mutant that produces AvrA protein caused *β*-catenin stabilization, Wnt2/Wnt11 increased expression, and IL-6, IL-4, IFN*γ*, and TNF*α* decreased expression [11, 12]. A different* in vitro *and* in vivo *study has shown that* Salmonella *SL1344 strain activated NF-*κ*B-dependent Wnt11/Wnt2 and IL-8 expression [10–12]. It is proposed that the deubiquitinase activity of AvrA inhibits *β*TRCP1-dependent ubiquitination of *β*-catenin and I*κ*B, the cytoplasmic inhibitor of NF-*κ*B. This AvrA inhibitory activity on ubiquitin ligases would make *β*-catenin more stable and the NF-*κ*B transcriptional activity less effective (Figure 2). Furthermore, ectopic overexpression of Wnt11 and Wnt2 decreased NF-*κ*B and AP-1 transcriptional activity [11, 12]. This evidence indicates that Wnt11 and Wnt2 trigger a negative feedback mechanism that controls NF-*κ*B activity through *β*-catenin activation.

## 3. *Citrobacter rodentium*: Crosstalk with the PI3K Pathway

Stabilization of *β*-catenin by enhanced phosphorylation at Ser552 has been observed in the intestinal epithelial cells from mice infected with the attaching/effacing (A/E) pathogen* C. rodentium* [16]. Mice treated with the PI3K inhibitor LY294002 decreased both the relative abundance of *β*-catenin phosphorylated at Ser552 and the expression of c-myc and cyclin D1. In contrast, IFN*γ* and TNF*α* expression in epithelial cells was increased after bacterial infection [17]. Interestingly, deletion of class IA PI3K gene in *IL*10^−/−^ mice showed impaired Akt and *β*-catenin signaling [18].

**Figure 3:**
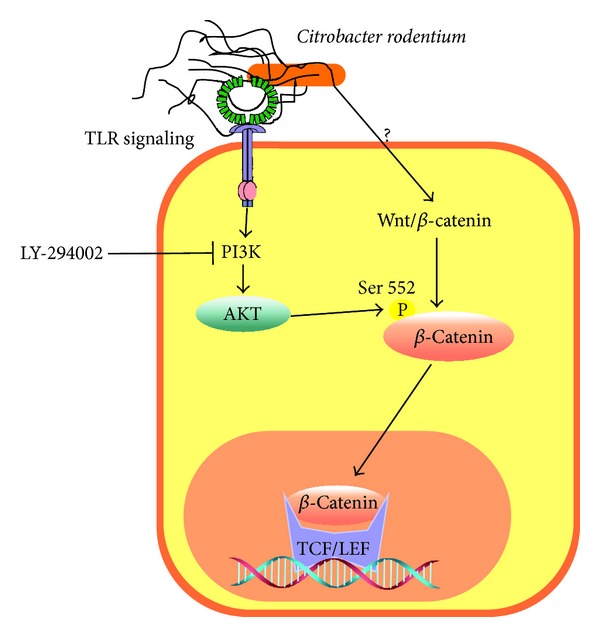
The PI3K/Akt signaling pathway enhances *β*-catenin activation. In colon epithelial cells infected with* Citrobacter rodentium*, Akt phosphorylates *β*-catenin at Ser552. The activation of *β*-catenin increases c-myc and reduces TNF*α* and IFN*γ* expression. An opposite effect is observed in cells incubated with the PI3K inhibitor LY294002.

GSK3*β* is a downstream effector of the phosphoinositide 3-kinase/Akt (PI3K/Akt) pathway as well as the Wnt/*β*-catenin* destruction complex *that negatively regulates *β*-catenin. The ability of GSK3*β* to promote or suppress the inflammatory response depends on the cell type and the stimulus [19]. In* C. rodentium *infections of CEC, Akt inhibits GSK3*β* by phosphorylation at Ser9 [20] with no phenotypic consequence reported so far. It is still not known yet whether this GSK3*β* phosphorylation depends on Fzd or TLR activation. Despite this lack of information, it is recognized that activation of the PI3K/Akt and Wnt/*β*-catenin pathways may cooperate to enhance *β*-catenin activity in* C. rodentium *infection mice models (Figure 3). In this context, it is also important to mention that in CEC infected with the Phop^c^ *Salmonella* strain, AvrA enhanced *β*-catenin phosphorylation at Ser552 by increasing Akt expression and Akt phosphorylation at Thr308 [21]. This evidence suggests that the PI3K/Akt signaling pathway cooperates to increase *β*-catenin activity in Phop^c^ *Salmonella *infections. However, whether AvrA directly alters PI3K activity, which is upstream of Akt, is not known. Future experiments should be designed to identify* C. rodentium *effector proteins responsible for *β*-catenin activation, which is also associated with CEC hyperplasia [20].

## 4. *Mycobacterium tuberculosis*: The Roles of Wnt Ligands and Fzd Receptors

**Figure 4:**
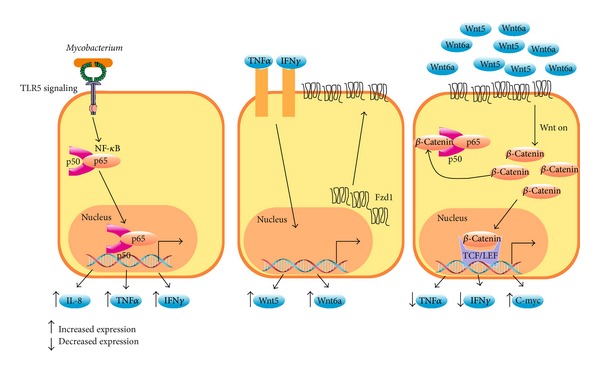
Frizzled receptors reduce inflammation. In epithelial cells and macrophages,* Mycobacterium tuberculosis *induces NF-*κ*B-dependent expression of IL-8, TNF*α*, and IFN*γ* (left panel) through TLR signaling. In turn, TNF*α* and IFN*γ* stimulation increases Fzd1, Wnt6, and Wnt5a expression at the membrane cell surface (middle panel). Finally, the binding of Wnt3a, Wnt5a, or Wnt6 to Fzd1 (right panel) induces stabilization of *β*-catenin, inhibiting the NF-*κ*B pathway. This interaction of *β*-catenin with NF-*κ*B decreases the expression of proinflammatory molecules such as TNF*α*.

*Mycobacterium tuberculosis *is able to interfere with some components of the Wnt/*β*-catenin pathway such as Fzd1, which is upregulated by TNF*α* and synergistically enhanced by IFN*γ* [8]. Presence of Wnt3a in the lungs of mice infected with* M. tuberculosis *affects the ability of macrophages to produce TNF*α* by increasing *β*-catenin transcriptional activity [8]. It is proposed that the upregulation of Fzd1 constitutes a mechanism by which Wnt3a represses inflammation, leading to a negative loop of inflammatory control in mice macrophages during* M. tuberculosis *infection (Figure 4).

Macrophages infected with* Mycobacterium bovis *show a robust increase in Wnt5a and the Notch1-target genes cyclooxygenase 2 (COX2), prostaglandin E2 (PGE2), and suppressor of cytokine signaling-3 (SOCS-3), which are involved in downmodulation of the inflammatory response. Interestingly, the effect on genes controlled by Notch1 was dependent on *β*-catenin and Jagged1, a Notch1 ligand [22]. It was also observed that activation of Notch1 by iNOS/NO and TLR2 signaling depended on *β*-catenin. These data indicate that *β*-catenin acts at late stages of infection to repress inflammatory programs triggered by TLR2 and iNOS/NO.

Interestingly Fzd receptors 2, 7, and 8 and Wnt ligands 11 and 2 are upregulated in CEC infected with* S. typhimurium *[11, 12] but not in macrophages infected with* M. tuberculosis*, in which Wnt5a and Fzd4 are highly expressed [22]. These data point out that expression of specific set of Fzd receptors and Wnt ligands depend on the pathogenic bacteria and the cell type. This hypothesis has been confirmed by Blumenthal et al. (2006) who showed that Wnt5a and Fzd4 were constantly expressed in macrophages but not in lymphocytes infected with* M. bovis* [23]. The TLR-NF-*κ*B pathway was responsible for Wnt5a expression and macrophages stimulated with Wnt5a produced IL-12p40 and IFN*γ*. Given that Wnt5a is able to activate *β*-catenin dependent and independent signaling, the role of *β*-catenin was not clearly established in this work. Recently, it was found that activation of the TLR-Myd88-NF-*κ*B pathway in foamy macrophage-like cells obtained from granulomatous lesion of mice infected with* Mycobacterium tuberculosis *induced the expression of Wnt6 [24]. Interestingly, Wnt5a induced an inflammatory response while Wnt6 decreased TNF*α* expression and triggered macrophage polarization toward an M2 phenotype [25, 26]. The fact that* Mycobacterium tuberculosis *induces *β*-catenin stabilization or Wnt5a expression may indicate a pathogenic mechanism whereby bacterial infection is successfully established. Other important and still unresolved issues refer to the* Mycobacterium *virulence factors responsible for regulating the expression of Wnt ligands and Fzd receptors and whether this regulation takes place in nonprofessional phagocytes.

## 5. *Pseudomonas aeruginosa*: Targeting the E-Cadherin Complex

Treatment of mouse corneal epithelial cells with LiCl, a compound that mimics Wnt/*β*-catenin pathway activation by inhibition of GSK3 activity, promotes host resistance against* Pseudomonas aeruginosa *infection [27]. Macrophages, neutrophils, and corneal epithelial cells infected with* P. aeruginosa *express proinflammatory cytokines IL-6, IL1-*β*, and TNF*α*, which are directly associated with the severity of the disease in a keratitis mouse model. In this model, *β*-catenin was degraded in a time-dependent mechanism. Furthermore, overexpression of *β*-catenin active mutants decreased proinflammatory cytokines, enhanced bacterial clearance, and reduced the severity of the disease caused by this bacterium [25, 26]. These data suggest that *β*-catenin degradation is required for higher expression of proinflammatory cytokines.

The quorum-sensing molecule acyl-homoserine lactone (AHL) from* P. aeruginosa *is able to disrupt the integrity of epithelial cell layer in Caco-2 cells by targeting *β*-catenin/E-cadherin complex. It was demonstrated that AHL directly interacts with *β*-catenin, resulting in the hyperphosphorylation of its tyrosine residues and translocation to the cytoplasm where it is phosphorylated by the* destruction complex *and degraded via the proteasome pathway [28]. Infection of mice urinary tract with* P. aeruginosa *strain PAO1, which is a producer of AHL and other quorum sensing molecules, results in high neutrophil recruitment and cytokine production compared with mutant strains unable to secrete this type of molecules [29].

*P. aeruginosa *also regulates the c-jun N-terminal kinases (JNKs) activity in corneal epithelial cells [26]. Interestingly, JNKs modulate adherent junction integrity by phosphorylating *β*-catenin at Ser33/37 and Thr41, the same residues that are phosphorylated by GSK3. However, it is not yet clear whether this JNK-dependent *β*-catenin phosphorylation induces its degradation [30]. Finally, it was also shown that corneal epithelial cells infected with* P. aeruginosa *express IL-6, IL-8, and TNF-*α* through a JNK-dependent mechanism [26]. These results suggest that* P. aeruginosa *targets *β*-catenin degradation to induce proinflammatory cytokine expression by secreting AHL and regulating the activity of JNKs.

## 6. *Helicobacter pylori*: Role of LRP6

The human pathogen* Helicobacter pylori *resides in the stomach of about half of the world population. The infection with this bacterium is associated with development of gastritis, peptic ulcer, and, in some cases, cancer. Although experimental data have shown that* H. pylori *induces activation of Wnt/*β*-catenin signaling through *β*-catenin stabilization in epithelial cells, a protein effector responsible for *β*-catenin stabilization has not yet been identified [21]. The cytotoxins VacA and CagA, expressed by virulent* H. pylori *strains, were not strictly required for *β*-catenin-induced stabilization [22]. Infections of human gastric epithelial cells NCI-N87 with* H. pylori *K1 induced phosphorylation of the LRP6 coreceptor at Ser1490 [31]. This phosphorylation was regulated by DVL2/DVL3 and was required for *β*-catenin nuclear translocation and transcriptional activity because knockdown of either of these two proteins reduced *β*-catenin-induced Axin2 expression. Moreover, evidence that LRP6 phosphorylation was dependent on a bacterial effector was based on experiments with* H. pylori *type four secretion system mutants or heat killed bacteria [31]. Nevertheless, no report so far has shown that stabilization of *β*-catenin by* H. pylori *influences the expression of inflammatory cytokines.

## 7. Concluding Remarks

The inflammatory response is a tightly regulated process because chronic or uncontrolled inflammation may cause tissue injury. Several signaling transduction pathways have been well characterized to induce inflammation; however, much less is known about the suppression and resolution mechanisms that control it. The evidence accumulated so far has pointed out that activation of the Wnt/*β*-catenin pathway reduces several molecular inflammatory processes that are triggered by bacterial pathogens. The fact that proinflammatory stimuli such as TNF*α*, IFN*γ*, and NO are able to increase the expression of Wnt/*β*-catenin signaling molecules indicate that these pathways should be interconnected. Also, it is likely that proinflammatory stimulation by bacterial infections is a requisite to activate Wnt signaling, indicating that *β*-catenin activity is required for late stages of the inflammatory response.

It is predictable that different specific combinations of Fzd receptors and Wnt ligands may promote or inhibit inflammation induced by bacterial pathogens. This is because bacterial pathogens modulate distinct key proteins that regulates Wnt *β*-catenin pathway and manipulates cell functions to increase its survival and spread invasion through different mechanisms. Future studies with different pathogenic bacteria and cell types will open new scenarios in which the knowledge of interconnection points in space and time of several signaling pathways may be used to design biotechnological approaches to resolve uncontrolled inflammation and enhance bacterial clearance.
